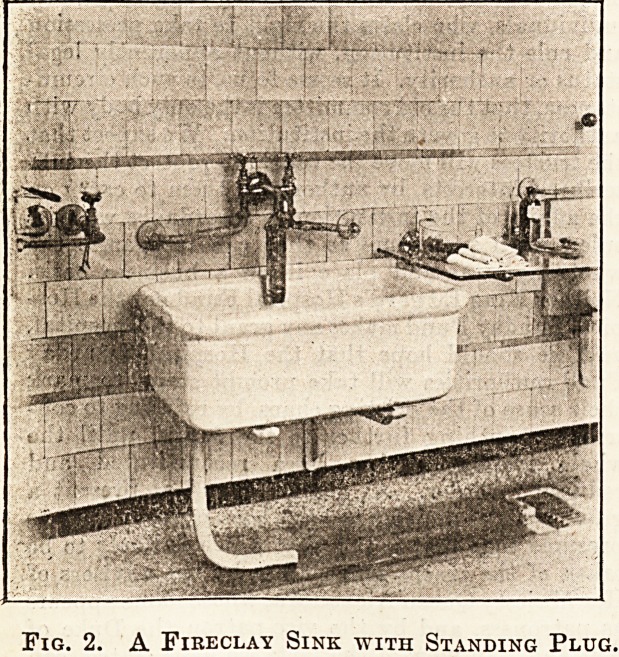# Admission Department of a General Hospital

**Published:** 1907-04-20

**Authors:** 


### ADMISSION DEPARTMENT OF A GENERAL HOSPITAL.

### 1. THE GATE HOUSE OR ADMISSION BLOCK. (*Concluded.*)

In the plan we published on page 46 last week, provision is made for a sitting-room for the medical officer on duty. This is a new and essential feature in the admission-block unit, for it should secure that no patient is kept waiting for many minutes before being seen. At present regrettable delays often take place, and much dissatisfaction and avoidable suffering may arise from this defect in the administration of a general hospital.

Fig. 1. A Fireclay Basin with an Arm Lever without Taps.

Fig. 2. A Fireclay Sink with Standing Plug.

Special attention should be paid to the plumber work; all pipes should be exposed, and all basins, sinks, and fittings of the simplest possible type. Fig. 1 shows a very good and simple basin made of fire-clay, suitable for an examination room or casualty operating room. The supply of hot and cold water to the basin is worked by means of an arm lever, and no taps are required. Another advantage is that the cold water always comes first, and there is no danger of being scalded.

Fig. 2 illustrates a serviceable type of sink, also made of fireclay, in which there is a standing waste acting also as a plug. The basins and sinks should discharge into an open gutter, protected at the end by means of a trap covered with a movable grating.

The other fittings in the examination-room should be as simple as possible, made of enamelled iron, and with ball-bearing casters to facilitate their being easily moved about.

### II. ITS THOROUGH SUPERVISION.

The superintendent should keep in direct touch with all that is going on and be satisfied that everything is in order; that courtesy and tact are extended to applicants for admission, as well as to their friends and to visitors to the hospital; and he should be ready to give advice or assistance to the junior medical officers whenever it is required.

The superintendent should be responsible for the admission and refusal of all patients; this, of course, implies the possession of a medical qualification. After the patients are admitted they are under the care and direction of the visiting medical and surgical staff, but no applicant should be refused admission without the superintendent's sanction, or, in his absence, that of his responsible deputy.

In a hospital connected with a teaching school, the number of attendants would necessarily be greater in proportion to the number of beds than in a hospital which is not a teaching school. But it must be borne in mind that a hospital of 800 beds does not require double the number of porters that a hospital with 400 beds requires; nor does a hospital of 400 beds require double the number of porters necessary for one of 200 beds. For a hospital which is a teaching school of, say, 500 to 550 beds, a janitor should have the assistance of six or seven porters. He is responsible to the superintendent for the work and good conduct of all the men under him; he sees that they are punctual and attentive to their duties, courteous to visitors, and careful in the handling of patients. He should also be responsible for all letters and parcels delivered at the institution, and see that they are regularly distributed. A note should be made if letters and parcels are opened or defaced when delivered, and the postman should endorse this. The admission department should never be left without a porter in attendance. In order that no confusion may arise, it should be clearly understood that when an ambulance attendant or policeman hands over the patient to the hospital staff, his responsibility ceases, and on no account should such an attendant be permitted to offer suggestions regarding the patient. (*To be continued.*)

## Figures and Tables

**Fig. 1. f1:**
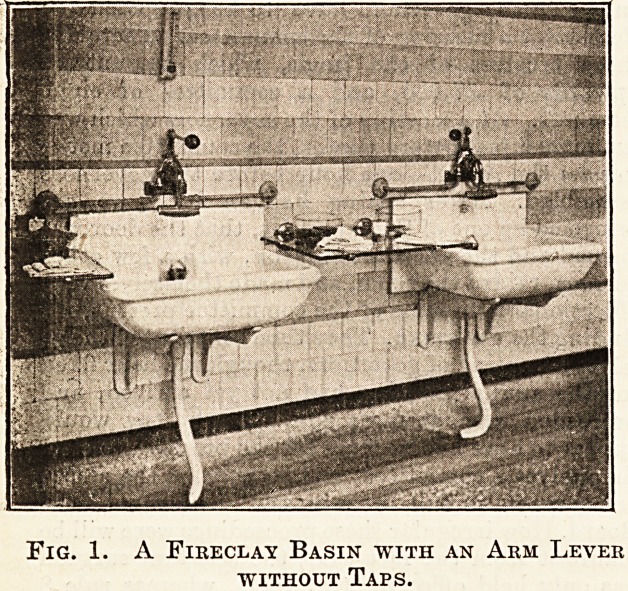


**Fig. 2. f2:**